# High mortality and prevalence of HIV and tuberculosis in adults with chronic cough in Malawi: a cohort study

**DOI:** 10.5588/ijtld.15.0388

**Published:** 2016-02

**Authors:** M. Nliwasa, P. MacPherson, M. Mukaka, A. Mdolo, M. Mwapasa, K. Kaswaswa, C. Msefula, G. Chipungu, H. C. Mwandumba, E. L. Corbett

**Affiliations:** *Helse Nord Tuberculosis Initiative, Department of Pathology, College of Medicine, Blantyre, Malawi; †Department of Public Health and Policy, University of Liverpool, Liverpool; ‡Department of Clinical Science, Liverpool School of Tropical Medicine, Liverpool, UK; §School of Public Health, College of Medicine, Blantyre; ¶Malawi-Liverpool-Wellcome Trust Research Programme, College of Medicine, Blantyre, Malawi; #Clinical Research Department, London School of Hygiene & Tropical Medicine, London, UK

**Keywords:** sub-Saharan Africa, epidemiology, presumed TB, human immunodeficiency virus

## Abstract

BACKGROUND: Adults with suspected tuberculosis (TB) in health facilities in Africa have a high risk of death. The risk of death for adults with suspected TB at community-level is not known but may also be high.

METHODS: Adults reporting cough of ⩾ 2 weeks (coughers) during a household census of 19 936 adults in a poor urban setting in Malawi were randomly sampled and age-frequency matched with adults without cough ⩾ 2 weeks (controls). At 12 months, participants were traced to establish vital status, offered human immunodeficiency virus (HIV) testing and investigated for TB if symptomatic (sputum for Xpert^®^ MTB/RIF, smear microscopy and culture).

RESULTS: Of 345 individuals with cough, 245 (71%) were traced, as were 243/345 (70.4%) controls. TB was diagnosed in 8.9% (16/178) of the coughers and 3.7% (7/187) of the controls (*P* = 0.039). HIV prevalence among coughers was 34.6% (56/162) and 18.8% (32/170) in controls (*P* = 0.005); of those who were HIV-positive, respectively 26.8% and 18.8% were newly diagnosed. The 12-month risk of death was 4.1% (10/245) in coughers and 2.5% (6/243) in controls (*P* = 0.317).

CONCLUSION: Undiagnosed HIV and TB are common among adults with chronic cough, and mortality is high in this urban setting. Interventions that promote timely seeking of HIV and TB care are needed.

## RESUME

CONTEXTE : Les adultes avec une tuberculose (TB) présumée dans les structures de santé d'Afrique ont un risque élevé de décès. La magnitude du risque de décès des adultes avec une TB soupçonnée au niveau communautaire n'est pas connue, mais elle est peutêtreélevée.

MÉTHODE : Les adultes signalant une toux ⩾ 2 semaines (tousseurs) lors d'un recensement à domicile de 19 936 adultes dans un contexte urbain pauvre du Malawi ont été sélectionnés au hasard et appariés sur l'âgeà des adultes sans toux ⩾ 2 semaines (témoins). A 12 mois, les participants ont été recherchés afin d'évaluer leur état de santé, de leur proposer un test pour le virus de l'immunodéficience humaine (VIH) et de rechercher une TB (crachats pour Xpert^®^MTB/RIF, microscopie de frottis et culture) en cas de symptômes.

RÉSULTATS : Ont été retrouvés 71% des tousseurs (245/345) et 70,4% (243/345) des témoins. Une TB a été diagnostiquée chez 8,9% (16/178) des tousseurs et 3,7% (7/187) des témoins (*P* = 0,039). La prévalence du VIH parmi les tousseurs a été de 34,6% (56/162) et de 18,8% (32/170) chez les témoins (*P* = 0,005) ; parmi les sujets VIH positifs, respectivement 26,8% et 18,8% ont été des diagnostics nouveaux. Le risque de décès sur 12 mois a été de 4,1% (10/245) chez les tousseurs et de 2,5% (6/243) chez les témoins (*P* = 0,317).

CONCLUSION : Une infection à VIH et une TB non diagnostiquées sont fréquentes parmi les adultes atteints de toux chronique, et la mortalité est élevée dans ce contexte urbain. Des interventions qui promeuvent la recherche précoce du VIH et la prise en charge de la TB sont nécessaires.

## RESUMEN

MARCO DE REFERENCIA: Los pacientes adultos con sospecha clínica de tuberculosis (TB) en los establecimientos de salud en África exhiben un alto índice de mortalidad. No se conoce esta tasa en las personas con presunción de TB en la comunidad, pero también podría ser alta.

MÉTODOS: Se practicó un muestreo aleatorizado de los adultos que referían tos de ⩾ 2 semanas de duración (tosedores) durante un censo domiciliario de 19 936 adultos en un entorno urbano de escasos recursos de Malawi y se emparejaron por frecuencia de edad con adultos que no referían este antecedente (testigos). A los 12 meses, se localizaron los participantes con el fin de verificar su estado vital, ofrecer la prueba diagnóstica de la infección por el virus de la inmunodeficiencia humana (VIH) y en caso de presentar síntomas, investigar la TB (muestra de esputo para la prueba Xpert^®^MTB/RIF, la baciloscopia y el cultivo).

RESULTADOS: Se localizó el 71% (245/345) de los tosedores y el 70,4% (243/345) de los testigos. Se estableció el diagnóstico de TB en el 8,9% (16/178) de los tosedores y el 3,7% (7/187) de los testigos (*P* = 0,039). La prevalencia de infección por el VIH en los tosedores fue 34,6% (56/162) y en los testigos fue 18,8% (32/170) (*P* = 0,005); la seropositividad frente al VIH en el 26,8% de los tosedores y el 18,8% de los testigos correspondió a un diagnóstico nuevo. El riesgo de muerte a los 12 meses fue 4,1% (10/245) en los tosedores y 2,5% (6/243) en los testigos (*P* = 0,317).

CONCLUSIÓN: La infección por el VIH y la TB no diagnosticadas son frecuentes en los adultos que presentan tos crónica y su mortalidad es alta en este entorno. Se precisan intervenciones que favorezcan la búsqueda oportuna de casos.

COUGH AND OTHER SYMPTOMS, including fever, weight loss, night sweats and haemoptysis, are used to define individuals with suspected tuberculosis (TB).^1^,^2^ Adults with suspected TB in health facilities in Africa have a high short-term (6–12 months) risk of death.^3^,^4^ The risk of death among adults with suspected TB at community level is not known, but may also be high and related to undiagnosed human immunodeficiency virus (HIV) infection and TB.

In Blantyre City, Malawi, HIV prevalence among the urban poor is high, at 18.5%,^5^ while TB prevalence is 0.9%.^6^ Malawi has implemented a highly successful antiretroviral therapy (ART) programme. HIV testing and counselling services have become increasingly available in health facilities in Malawi since 2010.^7^ By mid-2014, over half of the estimated 1 million people living with HIV in Malawi were aware of their status, almost 550 000 of whom were enrolled in ART or pre-ART care.^8^ HIV testing for all patients with TB symptoms is international policy and part of the national tuberculosis control programme (NTP), but this patient group has not been prioritised for provider-initiated testing and counselling in the 2015–2020 Malawi National HIV and AIDS Strategy, and the NTP policy has not yet been consistently implemented.^8–10^

The aims of the present study were to investigate vital status and the prevalence of diagnosed and undiagnosed HIV and TB among adult community members 1 year after enquiry about chronic cough in a household enumeration survey in urban Blantyre, Malawi. This study demonstrates the consequences of poor health seeking for cough and emphasises the need for combined HIV and TB screening at the household level in sub-Saharan Africa.

## METHODS

### Study design

This was a prospective cohort study of adults (age ⩾16 years) identified during a baseline household enumeration survey as part of a cluster randomised trial of intensified HIV-TB prevention in Blantyre, Malawi.^11–13^ In the parent trial, all adult household residents of 28 community health worker catchment areas were enumerated between March and August 2011, and these form the sampling frame for this study.

### Procedures at household enumeration

During household enumeration (first visit), a representative of the household provided demographic data and reported on the chronic cough status of all adults. Household members with reported chronic cough were not provided with individual patient management as part of the survey, although community-based TB and HIV testing services were introduced shortly afterwards and leaflets advising the investigation of chronic cough were left in all households. HIV testing was available to residents through an HIV self-testing intervention introduced in the study area in 2012.^11^,^13^ Adults with chronic cough had the opportunity of attending for routine care at two primary care clinics in the study area.

### Selection of participants

Random sampling with frequency matching by 10-year age bands was used to select a sample of enumerated household residents from two groups: household residents with chronic cough at enumeration (‘with baseline cough’) and household residents without chronic cough at enumeration (‘controls’). No other inclusion or exclusion criteria were applied to participants for this study.

### Procedures at second household visit

Twelve months after the baseline household enumeration, the research assistants made a second visit. With the help of community informants, the research assistants traced the dwellings of the participants using global positioning satellite (GPS) coordinates and descriptions of physical address recorded at baseline. For the second visit, participants were classified as lost to follow-up if they were not identified at their dwelling after three tracing attempts.

At the second visit, research assistants ascertained the participants' vital status and, where possible, interviewed surviving participants to determine whether they had chronic cough or had initiated anti-tuberculosis treatment in the previous 12 months. Questionnaires assessed exposure to biomass fuels for cooking or heating, exposure to occupational dusts (adapted from questionnaires by the Burden of Obstructive Lung Disease Initiative, BOLD^14^) and smoking.

Participants were screened for TB symptoms (presence of any of cough, fever, night sweats or weight loss). Participants reporting cough of any duration were asked to submit two sputum samples for TB diagnosis; samples were collected by research assistants and delivered to the research laboratory. Participants who reported cough were also asked to attend a hospital out-patient clinic and were offered transport reimbursement if they attended the clinic. HIV testing and counselling was offered to all participants, including those who reported that they had previously been diagnosed as HIV-positive, with referral to study HIV care clinics for ART assessments (CD4 and World Health Organization [WHO] clinical staging assessments) for those testing positive.

Death of a participant was confirmed by interviewing household members during the second household visit and by comparison with a list of deaths routinely collected by resident study community informants. Research assistants used the WHO verbal autopsy questionnaire to record the date of death and any clinical events or illnesses preceding death.^15^

### Case definitions for tuberculosis

Bacteriologically confirmed TB was defined as at least one positive sputum smear microscopy sample, a positive culture for Mycobacterium tuberculosis or a positive Xpert^®^ MTB/RIF (Cepheid, Sunnyvale, CA, USA) result. Clinically diagnosed TB was defined as report of anti-tuberculosis treatment without bacteriological confirmation, i.e., negative smear microscopy, culture and Xpert. TB cases were detected in two ways: by report of a participant registering for anti-tuberculosis treatment in routine care in the interval between household enumeration and the second household visit; and following TB screening during the second household visit.

### Laboratory methods

Sputum specimens were tested using fluorescence smear microscopy (Primo Star iLED^™^, Carl Zeiss Microimaging, Oberkochen, Germany) and Xpert, and underwent culture for TB using BACTEC^™^ MGIT^™^ 960 (BD, Sparks, MD, USA). Following Ziehl-Neelsen staining, acid-fast bacilli (AFB) positive cultures were confirmed as M. tuberculosis complex if the MPT 64 antigen test (MGIT TBc Identification test, BD) was positive, with microscopic cording and compatible growth characteristics at 37°C, at room temperature and at 45°C, and on Löwenstein-Jensen medium containing *p*-nitrobenzoate (PNB).

HIV testing was performed using the OraQuick ADVANCE Rapid HIV 1/2 Antibody Test (OraSure Technologies, Bethlehem, PA, USA) on oral fluid. The Determine HIV 1/2 test (Abbott Laboratories, Abbott Park, IL, USA) and Uni-Gold^™^ Recombigen^®^ HIV-1/2 test (Trinity Biotech, Wicklow, Ireland) were used to confirm positive results.

### Statistical analyses

The primary study outcomes were risk of mortality at 2 and 12 months after baseline enumeration, and prevalence of diagnosed and undiagnosed HIV and TB at the second household visit. For sample size calculation, we assumed a risk of mortality at 12 months after baseline enumeration of 5%^3^ among participants with baseline cough and 1%^16^ among controls. A total of 333 participants were needed in each group to detect this difference with 80% power, and α of 5%. We inflated sample size estimates by 20% to account for loss to follow-up; the final sample size was 400 participants in each group. Data were analysed using Stata 13.1 (StataCorp LP, College Station, TX, USA).

Baseline characteristics were compared between groups using the χ^2^, Fisher's exact or Wilcoxon rank sum tests. The risk of death was expressed as the proportion of adults who died at 2 and 12 months, respectively, and differences in mortality between groups were compared using the χ^2^ test. A Kaplan-Meier plot was used to depict survival in the two groups. Time to death was calculated as the time from the date of household enumeration to the date of death or censored on the date 12 months after household enumeration. Only participants with vital status ascertained were included in the analyses.

Risk factors for death in adults with baseline cough were investigated. These were demographic characteristics at baseline and interval HIV and TB status. Univariate analyses were performed to assess associations between covariates and the outcome. Variables that were significant on univariate analysis (*P* < 0.1) were included in the multivariate analyses. Sex and age were considered confounders a priori and were included in the multivariate model whether or not they were significant on univariate analysis.

### Ethical consideration

The study was approved by the College of Medicine of Malawi Research & Ethics Committee, Blantyre, Malawi. Written informed consent was provided by all participants (or household member if the participant had died) at the household follow-up visit.

## RESULTS

### Tracing of participants at second household visit

**Figure 1.**
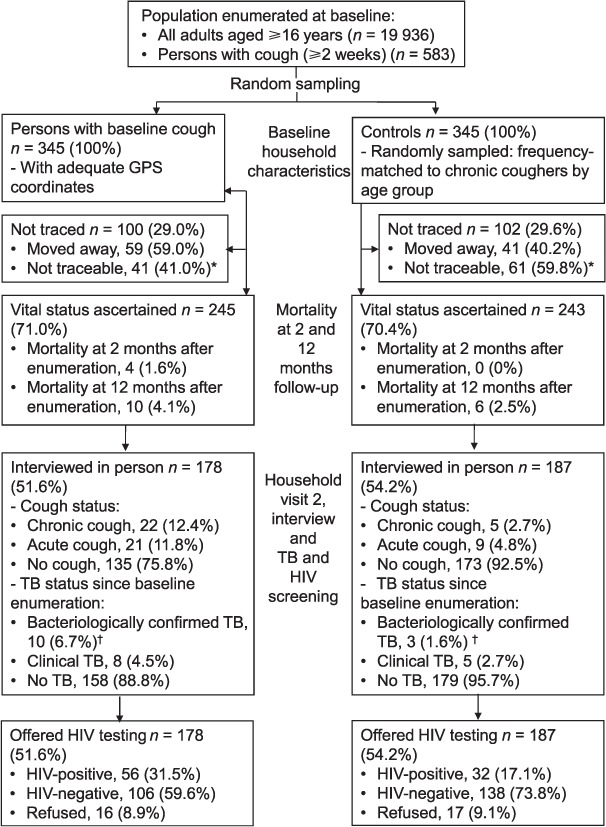
Recruitment and follow-up of participants. ^*^Participants were not identified as residents of the neighbourhood by household members or community informants. ^†^Bacteriologically confirmed TB was defined as at least one positive sputum smear microscopy sample, positive culture for Mycobacterium tuberculosis or a positive Xpert result. GPS = Global Positioning Satellite; TB = tuberculosis; HIV = human immunodeficiency virus.

The study population consisted of 19 936 adults, 583 (2.9%) of whom had reported chronic cough at household enumeration. Of the 400 participants with baseline cough who were sampled, 345 had sufficiently precise GPS coordinates to allow follow-up. Simple random sampling reduced the number of controls to 345 for parity with the number of participants with baseline cough (Figure 1). At the second household visit, 71% (245/345) of participants with baseline cough and 70.4% (243/345) of controls were identified. The reasons for unsuccessful tracing were participants moving away from place of residence or not being identified as residents of the study neighbourhood cluster by household members or community informants (Figure 1).

### Baseline characteristics

There were no significant differences in baseline sociodemographic characteristics between participants with baseline cough and controls (Table 1).

**Table 1:**
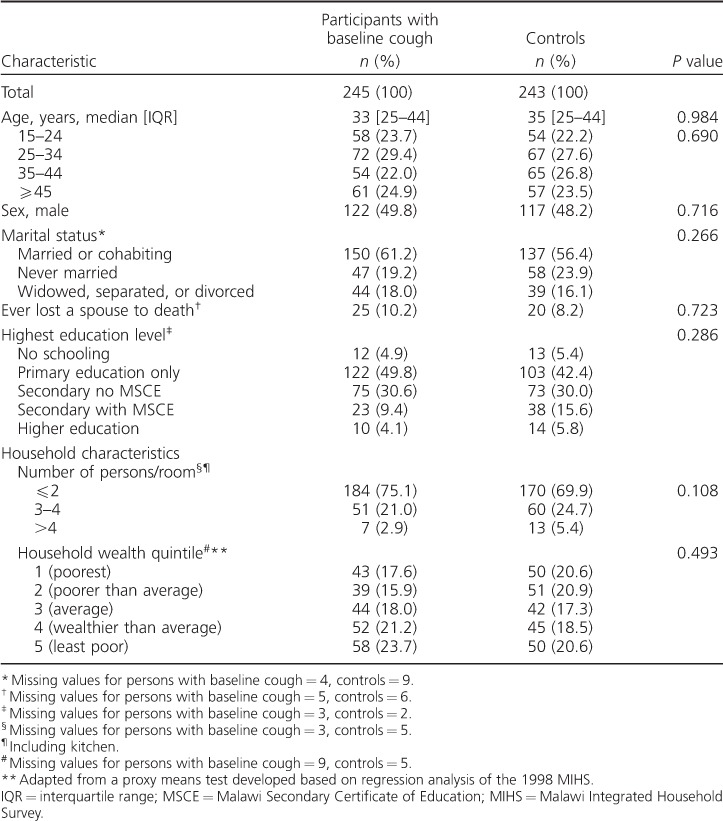
Baseline characteristics for participants with vital status ascertained

### Mortality at 2 and 12 months after baseline enumeration

**Figure 2.**
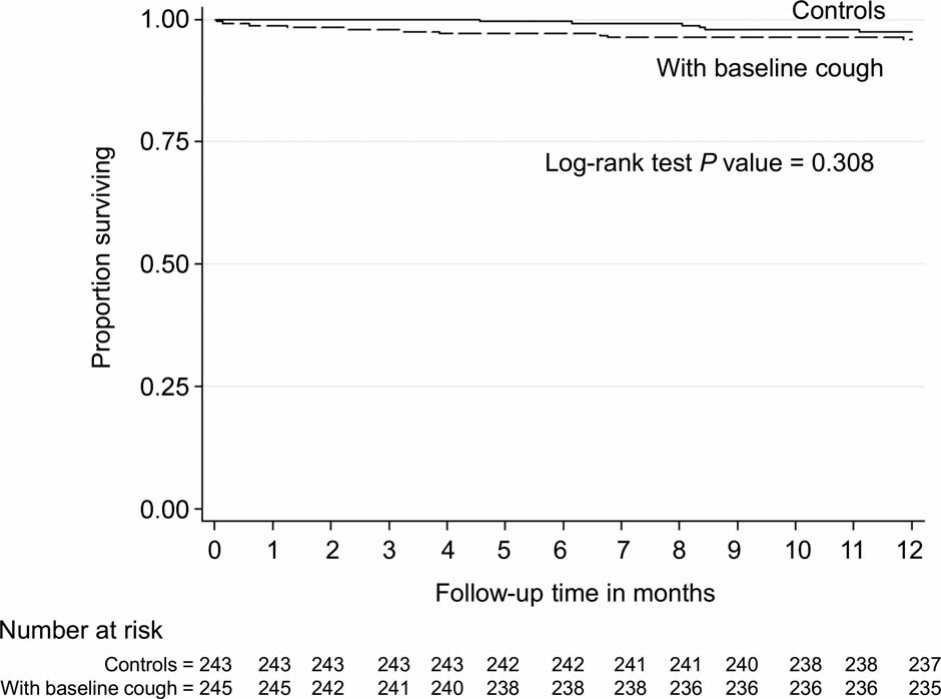
Kaplan-Meier plot of mortality in adults with baseline cough and controls.

At 2 months after baseline enumeration, all-cause mortality was 1.6% (4/245) among participants with baseline cough and 0% (0/243) in controls (*P* = 0.123) (Table 2). At 12 months, all-cause mortality was high in both groups: 4.1% (10/245) among participants with baseline cough and 2.5% (6/243) among controls (*P* = 0.317). Causes of death are presented in Table 2. Figure 2 shows Kaplan-Meier estimates of risk and timing of mortality.

**Table 2:**
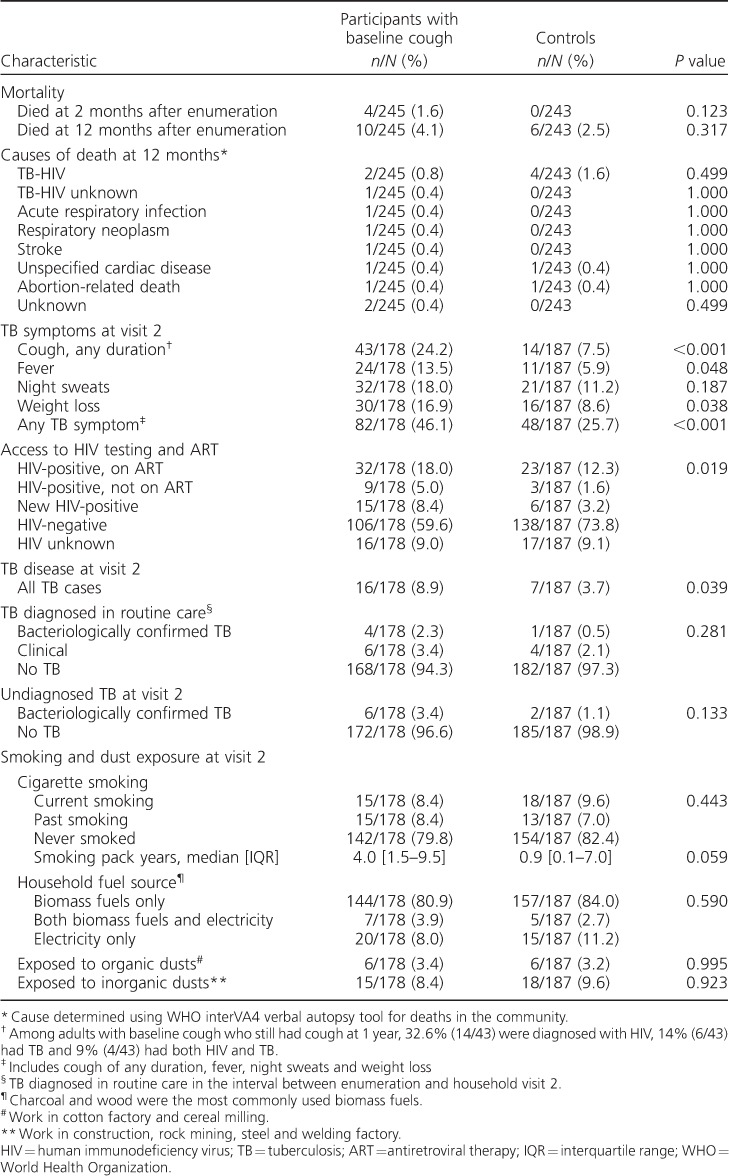
Mortality assessment, HIV and TB screening at household visit 2

### Risk factors for mortality among participants with baseline cough

Being diagnosed with TB was associated with a fourfold greater odds of death in the first 12 months after adjusting for age, sex and ever having ‘lost a spouse to death’ (adjusted OR 4.20, 95%CI 0.81–17.80, *P* = 0.082).

### HIV prevalence at second household visit

**Figure 3.**
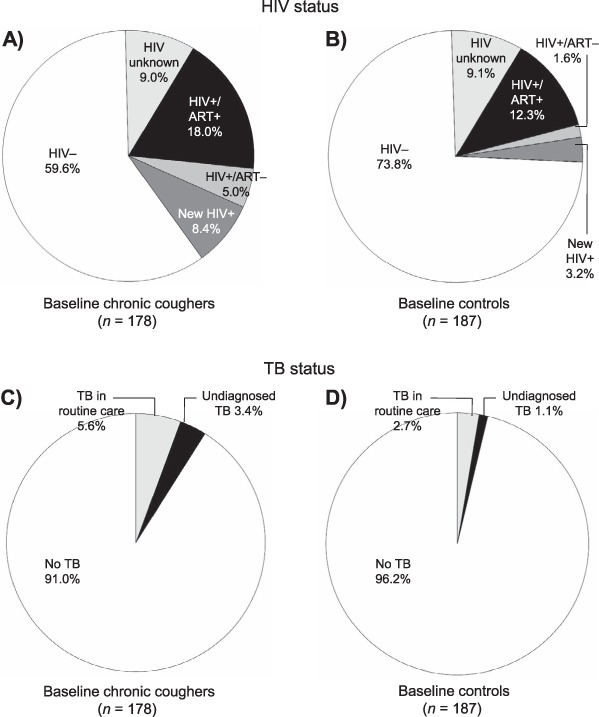
Care seeking for HIV and TB. In Panels A and B: HIV+/ART− = participants who knew their HIV status but were not on ART; new HIV = participants newly diagnosed as HIV-positive during screening at second household visit. HIV = human immunodeficiency virus; ART = antiretroviral therapy; + = positive; − = negative; TB = tuberculosis.

At the second household visit, the number of participants interviewed in person and offered HIV testing was 51.6% (178/245) among participants with baseline cough and 54.2% (187/243) among controls (Figure 1). Uptake of HIV testing was 91% overall. HIV prevalence at the second household visit was 34.6% (56/162) among participants with baseline cough compared to 18.8% (32/170) among controls (*P* = 0.001). Among those who were HIV-positive, 73.2% (41/56) of the participants with baseline cough and 81.3% (26/32) of the controls already knew their HIV-positive status, and respectively 57.1% (32/56) and 71.8% (23/32) were already on ART (Table 2 and Figure 3).

### Tuberculosis disease at second household visit

TB disease diagnosed in the interval between household enumeration and the second household visit was 5.6% (10/178) among participants with baseline cough compared to 2.7% (5/187) among controls (*P* = 0.281) (Table 2). The prevalence of undiagnosed TB at the second household visit was 3.4% (6/178) among participants with baseline cough compared to 1.1% (2/187) among controls (*P* = 0.133) (Table 2 and Figure 3).

## DISCUSSION

The main findings of this study were that there was a high burden of HIV and TB among adults with chronic cough and high 1-year mortality in both adults with chronic cough and controls.

**Table 3:**
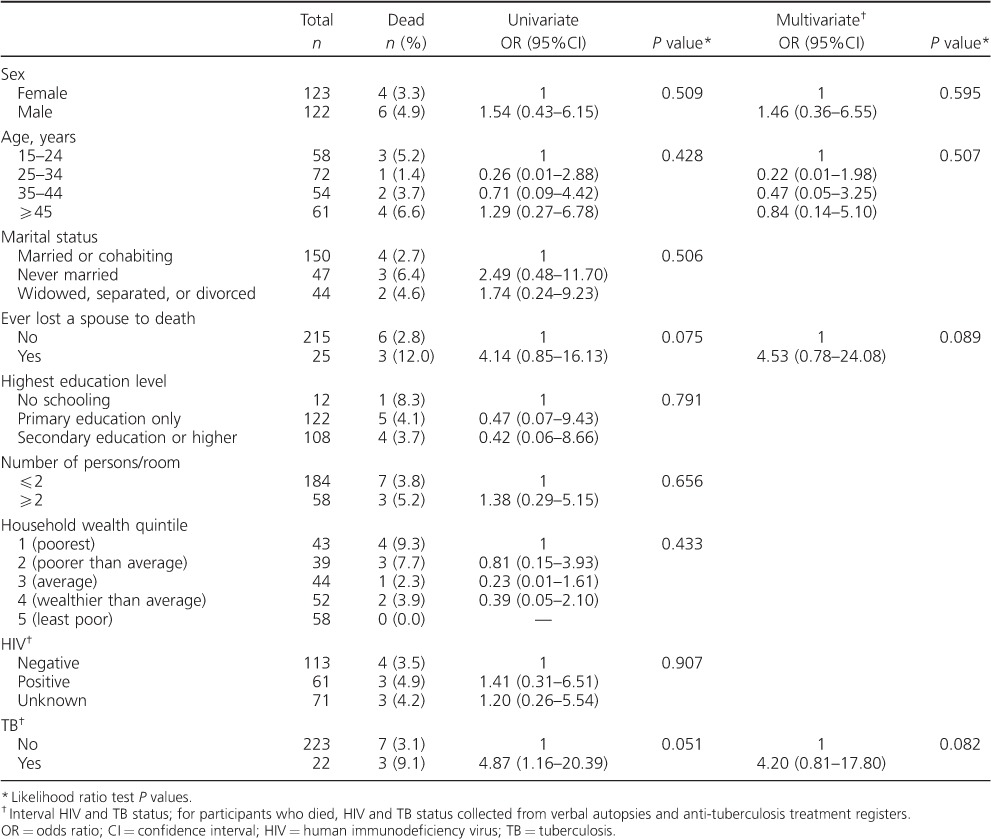
Risk factors for mortality at 12 months follow-up among adults with baseline cough

Mortality at 12 months follow-up was high even among adults without chronic cough at baseline. TB and ‘ever having lost a spouse to death’ were important risk factors for death among adults with chronic cough. HIV status was not ascertained at the baseline survey, however, and the finding that ‘ever having lost a spouse to death’ was associated with four-fold greater odds of death among adults with chronic cough suggests the probable role of HIV in driving this high mortality. HIV/acquired immune-deficiency syndrome accounted for 33.8–50.6% of all deaths among people aged 15–59 years in Africa, with TB being the main killer in people living with HIV.^17^

The Joint United Nations Programme on HIV and AIDS goal is that by 2020, 90% of all people living with HIV should know their status, and that 90% of these should be on ART.^18^ In the community studied, the proportion of those with HIV who knew their HIV-positive status was 73.2% among adults with baseline cough and 81.2% among controls. This represents both the achievements of the HIV programme in Malawi as well as the testing interventions by the local study, which provided access to self-testing for HIV.^5^,^11^ Notably, however, only 57.1% of HIV-positive adults with chronic cough were on ART, compared to 71.9% of HIV-positive controls. Both knowledge of HIV status and ART coverage were thus suboptimal, and more so in participants who reported chronic cough at baseline. There are probably multiple reasons for not initiating ART despite known HIV infection among our participants, including loss to follow-up before ART initiation despite eligibility.^19^ ART coverage at community level can be improved by introducing novel HIV interventions, for example HIV self-testing and home initiation of ART.^13^ ART initiation criteria are currently moving towards universal ART initiation, irrespective of CD4 cell count.^20^

Importantly, the diagnosis of TB was also suboptimal, with about a third of all TB cases in this cohort not having been diagnosed previously. Factors that contributed to delayed TB diagnosis and treatment include low disease awareness, use of informal care providers and stigma attached to HIV-associated TB.^21^,^22^ In Malawi, patients incur substantial costs when accessing TB diagnosis: poor patients spent an estimated 2.5 times their monthly income to obtain a diagnosis.^23^ Costs arose from fees and drugs during care seeking outside government facilities, transportation and food. In addition, patients lost an average of 22 days from work. The need to generate continuing income is a major barrier to timely access of TB diagnosis, especially among men trying not to miss work.^24^

There are a number of limitations to this study. The vital status of 30% of participants in each group was not ascertained at 12 months follow-up. However, loss to follow-up due to missed deaths was minimised by the presence of key informants who routinely conducted verbal autopsies. HIV screening and ART eligibility were not offered during the baseline survey; this was a missed opportunity both for facilitated access to care and for us to successfully investigate HIV status as a risk factor for death. Our ability to ascertain care-seeking pathways was limited by recall, given the long interval between the first and second household visits. Lung function assessment was not conducted, and causes of chronic cough other than TB were not thoroughly investigated.

## CONCLUSION

Mortality at 1 year was high both in adults with and in those without chronic cough, and the HIV and TB burden in adults with chronic cough was significantly higher, including undiagnosed disease. Chronic cough should be considered an important symptom for entry into HIV testing and care, and efforts must be made to provide HIV diagnosis for adults with symptoms of TB identified during population-level activities such as systematic TB screening or TB prevalence surveys.
